# Remote Moderator and Observer Experiences and Decision-making During Usability Testing of a Web-Based Empathy Training Portal: Content Analysis

**DOI:** 10.2196/35319

**Published:** 2022-08-03

**Authors:** Michelle Lobchuk, Prachotan Reddy Bathi, Adedotun Ademeyo, Aislinn Livingston

**Affiliations:** 1 College of Nursing Rady Faculty of Health Sciences University of Manitoba Winnipeg, MB Canada; 2 Department of Computer Science and Engineering Manipal Institute of Technology Manipal India; 3 Department of Electrical and Computer Engineering University of Manitoba Winnipeg, MB Canada

**Keywords:** web browser, user-centered design, qualitative research, internet, empathy

## Abstract

**Background:**

COVID-19 restrictions severely curtailed empirical endeavors that involved in-person interaction, such as usability testing sessions for technology development. Researchers and developers found themselves using web-based moderation for usability testing. Skilled remote moderators and observers are fundamental in this approach. However, to date, more empirical work is needed that captures the perceptions and support needs of moderators and observers in testing situations.

**Objective:**

The aim of this paper was to identify remote moderator and observer participant experiences and their use of certain tools to capture feedback of users as they interact with the web browser application.

**Methods:**

This research is part of a broader study on an educational web browser application for nursing students to learn perspective taking and enhance their perceptual understanding of a dialogue partner’s thoughts and feelings. The broader study used a quantitative and think-aloud qualitative problem-discovery usability study design. This case study explored written accounts of the remote moderator and observer participants regarding their roles, experiences, and reactions to the testing protocol and their suggestions for improved techniques and strategies for conducting remote usability testing. Content analysis was used to analyze participants’ experiences in the usability testing sessions.

**Results:**

We collected data from 1 remote moderator and 2 remote observers. Five themes were identified: dealing with personal stressors, dealing with user anxiety, maintaining social presence, ethical response to the study protocol, and communication during sessions. The participants offered recommendations for the design of future remote testing activities as well as evidence-informed training materials for usability project personnel.

**Conclusions:**

This study’s findings contribute to a growing body of endeavors to understand human-computer interaction and its impact on remote moderator and observer roles. As technology rapidly advances, more remote usability testing will occur where the knowledge gleaned in this study can have an impact. Recommendations based on moderator and observer participant perspectives identify the need for more evidence-informed training materials for their roles that focus on web-based interpersonal communication skills, execution of user testing protocols, troubleshooting technology and test user issues, proficiency in web conferencing platforms, behavior analysis and feedback technologies, and time management.

## Introduction

### Background

#### Moving Toward Remote Usability Testing

Usability testing is a fundamental step for evaluating and developing quality technologies, products, or services [1]. Usability testing involves the systematic evaluation of how effective, efficient, and satisfactory a product or service is when a user interacts with it [2]. This testing helps developers identify and refine the product or service before full-scale uptake by potential future users or implementation in the marketplace. Since the COVID-19 pandemic, in-person testing sessions for technology development have been curtailed. Remote usability testing has become the default approach to maintain advancements in technology evaluation or research [3]. Many system developers, usability personnel, and researchers have resorted to web-based moderation for remote usability testing, which is the focal context of this paper.

Remote usability testing was introduced 20 years ago as a means of conducting usability assessments of participants or users who were in separate locations or separated by time from the researchers or practitioners [4,5]. Since that time, a range of web conferencing platforms have emerged, and an array of features and functions are now available that can advance remote usability testing (eg, Microsoft Teams, Zoom, Skype, and Cisco Webex). Hill et al [5] provided an overview of the different types of remote usability testing methods and a summary of key findings about their use with users. Across these different methods, skilled remote moderators and observers are essential.

The literature on remote usability testing is evolving in an emerging field [5]. Gaps in knowledge still exist regarding the method. Wozney et al [6] were among the first authors to highlight the empirical gap in the influence of moderators and observers on remote web-based usability testing. The empirical literature has instead tended to focus on the feasibility, logistics, and pros and cons of the method. The rapid review of studies by Hill et al [5] found benefits to remote usability testing, such as facility attendance not being required and being more convenient and affordable for research personnel and participant users, the ability to more easily recruit a broad and diverse participant sample, and the opportunity to use technology more realistically in the participant user’s own environment. The disadvantages were the loss of contextual information, challenges in detecting nonverbal cues, and dealing with technology network issues [5]. Other factors that can influence the efficiency, validity, and reliability of remote usability testing and are difficult to control include user characteristics, internet speed, devices used, pace of the testing session, technical skills of participant users, and unforeseeable disruptions or interruptions [5].

Of note, Molich et al [7] explained that best practices or guidelines for remote moderation and observation are often derived from experiential evidence that is published in books and websites. However, *human factors* associated with the moderator and observer role are receiving increasing attention as salient influences on the “quality” of usability testing results. Such factors can include the moderator’s cognitive load, communication challenges, and handling of multiple technical issues that affect their role and social presence and, thus, participant users’ performance outcomes [6]. Wozney et al [6] identified these as “sociotechnical human factors,” including the attentional divide between auditory, textual, and visual stimuli. In testing sessions, these factors are dynamic and put cognitive demands on remote moderators and observers. In an earlier article, the impact of social context on performance outcomes was reviewed by Trivedi and Khanum [8]. These authors identified the social context or social environment as comprising people (eg, participant users, research personnel such as evaluators, and other people) whose presence during testing can have a substantial role in usability evaluations. However, to date, social context and human factors linked to the moderator and observer roles have not received the same attention as the physical context (ie, laboratory and field settings) as potential moderators or mediators of usability testing outcomes [6,8].

#### The Remote Moderator Role

The remote moderator is pivotal in usability testing. This role requires skilled, simultaneous execution of the following tasks: knowledge and use of technology and its features or functions; observation skills in watching the user interact with the product; careful documentation of usability issues; good communication skills that include ad hoc questioning, probing for clarity, and verbal and nonverbal communication; careful listening to the user’s spoken-out-loud thoughts and feedback; and knowing when to take the lead in asking the participant to perform tasks and when to stay quiet as the user engages in the tasks. The goal is to build trust with the user and obtain honest feedback about the product or service and the user’s experiences as well as foster user motivation to complete the assigned tasks. Wozney et al [6] explained that these skills are similar to those used by qualitative researchers. Others have stated that these skills affect the quality of the usability testing findings and user feedback [7].

#### The Remote Observer Role

Most of the time, the literature tends to focus on the moderator role [7]. However, considering the multitude of simultaneous tasks required of the moderator, it makes sense to include a “silent” observer or note-taker in the testing sessions. The observer or note-taker can serve as a vital second pair of eyes and ears in witnessing how the user interacts with the technology, product, or service. Tullis and Albert [9] described how the observer or note-taker has better “behind the scenes” opportunities than the moderator does to observe participants directly (eg, what they are doing, where they struggle, and how they succeed) and quickly record the user’s performance (eg, recording the time to complete tasks). The early stages of the design and development process often involve small sample sizes (eg, 5 to 6 participants) to identify issues with the design and administer fixes to those issues. Regardless of sample size, it is plausible that involving both an observer and a moderator could help lessen the odds of missing major usability issues in comparison with when the busy moderator needs to observe alone.

In summary, while conducting a review of the remote usability testing literature for the broader project, we discovered studies that reported on “how to” support the moderator and observer roles [7,10,11]. Other authors [5,6] made a call to action to better comprehend complex linkages among human factors (eg, social context or environment or “who” is present at testing), human-computer interaction, and web-based technology and tools to advance the remote usability testing approach. More peer-reviewed research needs to focus on moderator and observer subjective viewpoints regarding their influence and related factors for successful usability testing efforts (eg, motivating users to engage with tasks, obtaining quality feedback from users, and efficient decision-making about priority design refinements).

### Study Aim and Research Question

Our broader project involved usability testing of an educational web browser application (called In Your Shoes) for nursing students to learn perspective taking and enhance their perceptual understanding of a dialogue partner’s thoughts and feelings. The aim of this study was to present an adjunct component of this broader project, where the experiences of 1 moderator and 2 silent observer participants during remote usability testing sessions were uncovered. The qualitative research question was “What are the perceived experiences of the lead remote moderator and 2 silent remote observers using web conferencing and various tools to capture user feedback on the empathy application during remote usability testing?”

## Methods

### Research Design and Usability Testing Context

A single-case methodology was used as an adjunct to a broader study that used a quantitative and think-aloud qualitative problem-discovery usability study design. A report on the quantitative and qualitative user feedback will be provided elsewhere. In this paper, we report only the experiences of the remote moderator and remote observers. The case method approach allowed us to closely explore the experiences of the lead remote moderator and 2 silent remote observers using a web conferencing platform during their remote testing of the empathy web training portal with nursing student users [12]. The “case” was the lived experience of “how” the remote moderator and remote observers executed the study protocol and “why” they had responses to certain “live” events. The “context” of the lived experiences was the protocol-driven usability testing sessions [13]. We collected and content-analyzed their written responses to a list of questions about their experiences in their roles and reactions to the usability testing protocol.

### Ethics Approval

This study was conducted remotely with participant users in a midprairie province city in Canada. This study received ethics approval HS24965 (R1-2021-082) from the University of Manitoba Research Ethics Board and access approval from the College of Nursing. The remote moderator obtained informed consent from users before commencing the study protocols with them.

**Figure 1:**
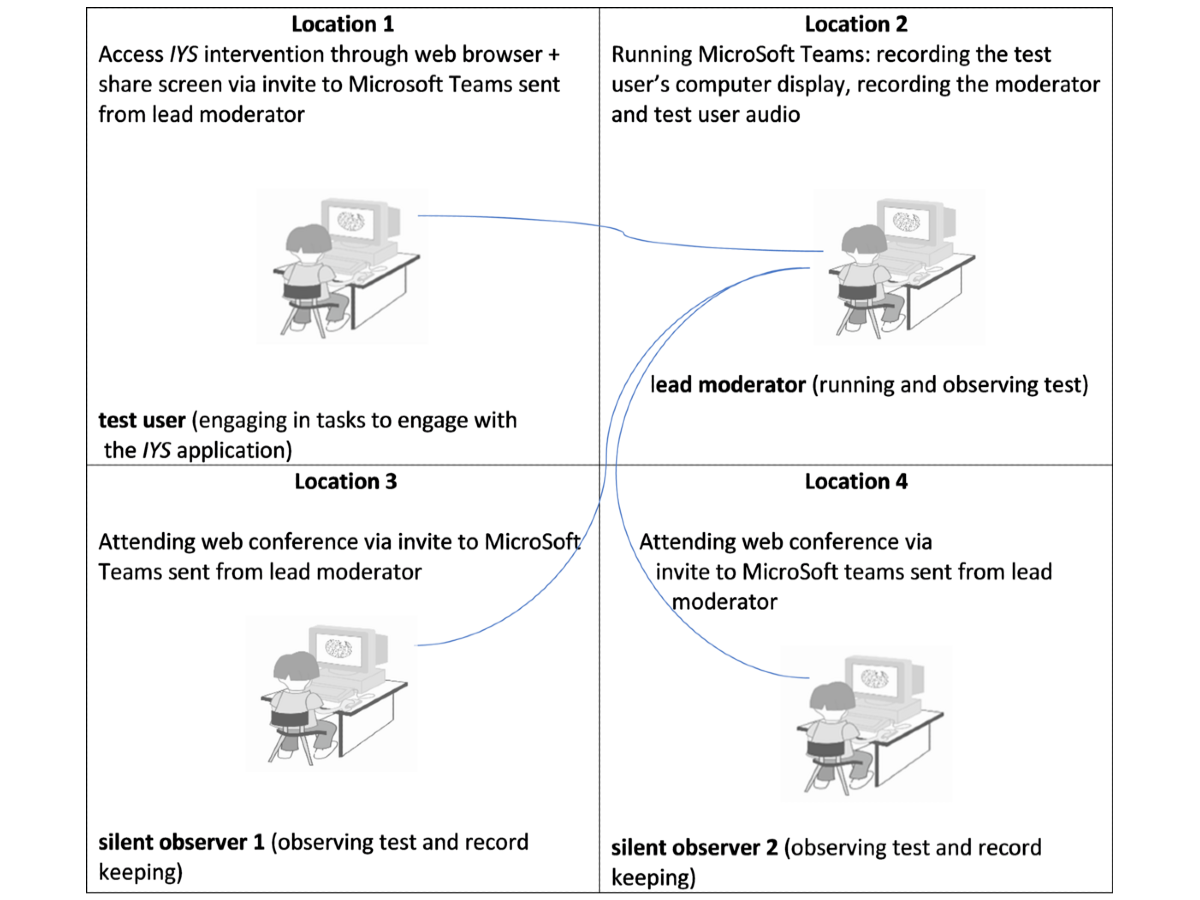
Web conferencing test environment set up for phases 1 and 2.

The broader study consisted of 3 consecutive phases. Each phase was interspersed with elements of data analysis and iterative design refinements for application prototype redevelopment [9,14]. A summary of the usability testing setup is provided in Multimedia Appendix 1. Figure 1 depicts the usability testing session scenarios for phases 1 and 2 and the roles. Phases 1 and 2 entailed a 1-hour video recording as moderated by the remote moderator. The remote moderator guided users to engage in tasks followed by immediate content analysis by the principal investigator (ML), the remote moderator, and the remote observers of the transcriptions of the users’ speak-aloud responses and video-recorded “performance” of tasks. Before starting our usability testing protocol, ethics approval was obtained from our institutional research ethics board, and access approval was obtained from the College of Nursing.

### Characteristics of the User Sample

#### Undergraduate Nursing Students

We aimed to recruit 12 undergraduate nursing students from 1 university. The sample size was based on sampling and recruitment for usability studies to maximize the expected level of problem discovery [9,14]. Owing to the small number of student volunteers (n=8) with constrained schedules to participate freely, random assignment to each phase was not feasible. Instead, the remote moderator selected a combination of second- to fourth-year male and female students from the undergraduate baccalaureate program in nursing who owned or had access to a PC desktop computer, an Apple desktop computer, or a tablet device for 3 cohorts of a total of 8 students (phase 1: n=3, 38% of students; phase 2: n=3, 38% of students; and phase 3: n=2, 25% of students).

#### Recruitment

All second- to fourth-year students received an initial email invitation from our unit’s research office on behalf of ML that was followed by 2 email reminder invitations. Interested students contacted the remote moderator, who emailed them an information package about the usability testing sessions. At a convenient time, the remote moderator conducted a video or phone call with potential users to further explain the study and expectations during the testing sessions. The remote moderator’s script was as follows: “We are testing the application to get user feedback, so we will have a video call where I will be joined by 2 other research assistants. They will be silent observers, so they will have their cameras and microphones off. During the call, I will get you to share your screen and see how you interact with the application; I will prompt you to answer questions and ask you to do tasks on occasion. We really want to get insight into the user experience, so we want honest feedback about the thoughts and feelings of the users. The video call will be about an hour long, give or take 15 minutes.”

### Setting, Access, and Preparation for the Usability Testing Sessions

#### Overview

An undergraduate research assistant from the computer sciences served as the lead remote moderator from her isolated setting with no distractions. The remote moderator facilitated the testing sessions using a functioning microphone and the institutionally adopted Microsoft Teams screen- and audio-sharing tool that allowed student users to share their screens. Remote observation via screen sharing, aided by Microsoft Teams and video files viewable in Microsoft Teams, helped the team capture and review behaviors and narrations as the users performed the tasks. In total, 2 undergraduate research assistants in computer sciences and computer engineering served as silent remote observers who watched and recorded user behaviors during tasks from their respective isolated settings. The remote observers also performed application refinements based on user input.

A member of our investigative team (Yumiko Sakamoto) was an expert in human-computer interaction who trained the remote moderator and remote observers on how to conduct a 1:1 session with users. The training included how to observe and keep records in Microsoft Excel, prompt users to speak aloud to explain the actions they were taking, and conduct user exit interviews. The users were not provided with the task list to review before the scheduled session. Instead, at scheduled sessions, the remote moderator provided users with realistic task scenarios to interact with the application interface and perform tasks in the sequence of steps that they would need to carry out if they were using the application in a real-life setting (ie, set up an account, open training documents, upload an mp4 video, engage in video tagging, and obtain their perceptual understanding score) [15].

#### Pilot Test

The first step was to conduct pilot-testing sessions using Microsoft Teams that were remotely moderated with 2 nursing student users at the end of June 2021 and the beginning of July 2021. The pilot sessions helped the remote moderator and remote observers become acquainted with each other, determine the best approach to conduct the testing sessions collaboratively, and identify issues related to moderating sessions (eg, avoiding asking leading questions and the need to send participants a video to upload in advance of the testing session). The remote observers also identified that the performance metrics tool required reformatting based on the anticipated order of the user tasks to be performed. On the basis of pilot test user feedback, the remote observers made application refinements (eg, provided clearer explanations of key features directly on the application pages, inserted colored text to delineate application “taggers,” and fixed glitches with log-in and sign-out functions). Commencing in mid-July 2021 until the end of August 2021, 3 phases of user testing sessions were successfully executed at the College of Nursing.

### Description of the Web Browser Application

We developed and tested the first prototype of an empathy-related video feedback intervention (*In Your Shoes*) to improve perspective-taking skills and perceptual understanding by clinicians of their clients (or accurate understanding of another person’s thoughts and feelings) that requires attendance in a laboratory setting plus a desktop, a stationary camera, and a commercial video analysis software program [16,17]. Our *In Your Shoes* intervention was adapted from the award-winning research in social psychology on empathic accuracy (or the extent to which an individual’s inferences of the content of another person’s thoughts and feelings are accurate) by Dr William Ickes et al [18,19]. The work by Ickes provided us with a practical, reliable, and objective method for measuring an individual’s ability to accurately infer another person’s thoughts and feelings during a video-recorded interaction.

To enhance accessibility and ease of use, our investigative team and the Red River College Polytechnic ACE Project Space students transformed the existing in-laboratory desktop approach into a unique web browser application version to learn perspective taking on any computer or tablet device. The development process consisted of the front end, server, database, and cloud storage system as the 4 key interconnected system components required to deploy the In Your Shoes web application. The front end retains the user’s interface (ie, everything that the user sees is rendered here) and was developed using the React framework (Meta). The data displayed on the front end are stored mainly in MongoDB (MongoDB Inc), which houses data including user information, tags, and video data. The videos are stored on Amazon Web Services using an S3 bucket, whereas links to the videos are stored in MongoDB. For the front end to use and display the data from the database and cloud storage system, the Django Representational State Transfer framework (Django Software Foundation) is used on the server to structure the data in a way that is easily accessible from the front end. Essentially, the data are taken from storage, structured on the server, and rendered on the front end so that the user can interact with them (see Multimedia Appendix 2 for the system components). The application was developed to replicate the existing in-laboratory process with no special requirements for hardware. The only hardware needed is a computer, laptop, or mobile device with a functioning browser and internet access. To secure evidence on prospective users’ experiences with the *In Your Shoes* application features and functions and to obtain feedback for further application refinements, we conducted a usability testing project with the remote moderator and silent observers who were the focus of this case study.

### Study Procedure

#### Remote Moderator Role, Responsibilities, and Recommendations for the Broader Study

The remote moderator was responsible for facilitating the remote testing sessions, answering user questions during testing sessions, managing the virtual video recording and transcription platform, and editing the transcripts, as well as for data collection, data analysis, and data management via the Qualtrics (Qualtrics International Inc) and Microsoft Teams platforms. After the study’s expectations were explained, the remote moderator sent users the Qualtrics link to the informed consent and demographic data form for phases 1 and 2. Users in phase 3 were sent the Qualtrics link to the consent form and demographic data form plus the usability tool. A total of 24 hours before the scheduled session, the remote moderator sent instructions to download the Microsoft Teams application and an mp4 video to upload during the testing session. Once the consent and demographic forms were signed, the remote moderator sent a Microsoft Teams calendar invitation to the user and remote observers for the testing session. Approximately 5 minutes before the video call, users were sent the link to the In Your Shoes application. There was 1 participant who did not attend the initial or rescheduled sessions. In addition, 2 other students signed their consent forms but never responded to the remote moderator’s email requests to schedule a testing session. A recommendation is for future users to send out several reminders (ie, 48 and 24 hours in advance of the scheduled testing session).

Once the user was logged into the session, the remote moderator immediately thanked them for participating. A repeated explanation of the study expectations was provided. The remote moderator encouraged users to be honest with their feedback. Despite having sent students a reminder to download the Microsoft Teams application in advance of the session, most students used the browser application instead. Regarding users’ computer technology skill level and experience with Microsoft Teams, some had used it before, but most had not. This lack of experience resulted in the remote moderator needing to explain to them how to share their screens. During the testing session, the remote moderator learned that the desktop and browser versions of the Microsoft Teams functions and features were different. This became an issue when the remote moderator would explain how a user could share their screen and the user was using the browser instead of the desktop version. In that case, the remote moderator was challenged to help them as the user did not have access to the same buttons as the remote moderator, who used the desktop version. For the few users who had a very slow internet connection, the video quality of the call was fine, but it would take a while to upload a video to the application for the video-tagging exercise. While they were uploading the video, the remote moderator would either perform another task with the users or just wait for the video to be uploaded. Otherwise, the remote moderator and remote observers found Microsoft Teams intuitive to use. The recording and transcription facilities were extremely helpful in capturing session data, which allowed them to quickly rewatch the sessions to ensure the accuracy of the data that they had collected.

During phase 1 and phase 2 sessions, the remote moderator encouraged the user to engage with the application as if they were alone with it. Allowing the user to figure things out on their own was the most useful approach. The most information about user interaction with the application came from observing the user and asking them questions about their actions instead of telling them how to perform the task. The strategy used was to wait for the user to interact with the web application and remind them to speak out loud while they were doing an activity. Asking the user to speak aloud was helpful when editing the transcripts wherever users were unclear and when adding context to the testing session. It was easier to determine what was happening when the user described the webpage they were interacting with during the session.

For phase 3, users engaged with the application in their own environments for 1 week, and they were not observed. The remote moderator sent them detailed instructions about the purpose of the study and specific tasks for them to complete. The users emailed the remote moderator to express their confusion about uploading a video that they were asked to create based on a conversation they were asked to have with a family member or friend. Owing to pandemic restrictions, both users explained that they could not locate a family member or friend that they felt comfortable with to do a video-recorded dialogue and then upload and analyze this video recording in the application. Testing the independent use of these tasks (ie, the main functions of the application) was not possible.

Once the users had engaged with the application for 1 week, a scheduled 60-minute video-recorded and scripted interview was led by the remote moderator, who administered the usability tool to capture user “satisfaction” with the *In Your Shoes* application [20]. A total of 3 questions were added to capture language understandability, visual-interactional appeal, and whether use expectations were met. A think-aloud process was used where the remote moderator asked users to speak aloud their thoughts as they completed each statement on the usability tool [21]. The remote moderator used probes such as “I would like to hear what you were thinking when you answered this question.” Users also provided information on the type, brand, and operating system of the device used; the number of times they accessed the application; and the amount of time they spent on the application each time they accessed it over the previous week.

#### Remote Observer Roles, Responsibilities, and Recommendations for the Broader Study

Silent remote observation was an important aspect of phases 1 and 2. In total, 2 remote observers monitored how each of the participants traversed through the application and maintained a record of (1) the user’s test environment, (2) paths that users navigated to perform a task, and (3) temporal and cognitive resources each user spent when performing a task. Once the remote observers had reviewed the data collected in the usability testing session, they performed refinements to the application to make the user experience better for the subsequent phase.

**Figure 2:**
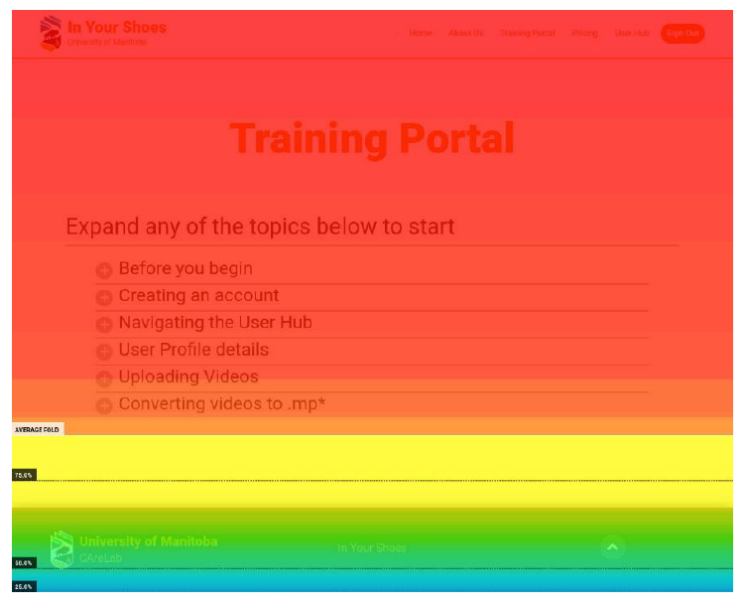
Hotjar (Hotjar Ltd)—scrolling activity (on the application’s Training Portal for a sample of 282 scrolling activities; red indicates that all or almost all users have seen this part of the page).

**Figure 3:**
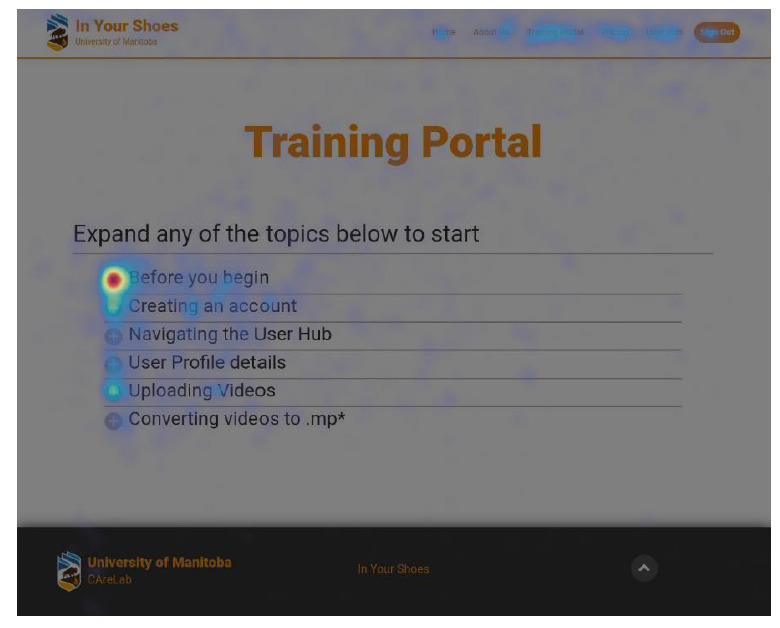
Hotjar (Hotjar Ltd)—clicking activity (on the application’s Training Portal for a sample of 175 clicks).

For phases 1 and 2, the remote observers developed a predefined protocol, as guided by Tullis and Albert [9] and Nielsen [14], to capture performance metrics on predefined tasks that the users had to perform. The performance metrics were recorded by the remote observers as they monitored an ongoing testing session. The remote observers used 2 electronic forms with Google Forms that supported a wide range of inputs (Multimedia Appendix 3). The think-aloud method used by the remote moderator with the users helped the remote observers identify technical issues and required refinements to the application [22]. One remote observer recorded the success of the user in completing predefined tasks and some remarks regarding how well they completed the tasks. The other remote observer recorded the number of mouse clicks required for the user to complete each task. This proved to be challenging at times depending on the device used by the user. Some users had mouse devices that were inaudible, which made it difficult for the remote observer to see and hear the clicks to capture them in the performance metrics. In addition, there were occasional issues with choppy video depending on the user’s internet connection. Hotjar (Hotjar Ltd) with heat maps was also used as a behavior analytics and feedback data tool (see Figure 2 for scrolling activity and Figure 3 for clicking activity). This tool helped the project team understand what the users were doing on the website pages (eg, where they clicked or scrolled and what they looked at or ignored) and then identify priority application refinements. The performance metrics spreadsheets were stored on Google Drive, which made it easy for the team to manage access and collaborate in evaluating the results. Overall, there was great benefit to having the remote observers directly monitor and record the impact of their implemented changes when different users interacted with the application across the phases.

The remote observers were also the “application fixers” who were able to quickly and accurately identify required application refinements by directly communicating with the users at the end of the usability testing sessions. This dialogue summarized how well the remote observers felt the user did during the session and allowed the remote observers to ask the user about further suggestions for application refinements. The remote observers participated in testing sessions at their physically distanced work stations in remote settings where external disturbances were limited; this fostered their focused observation and accurate data collection. Unforeseen disruptions did arise occasionally during remote testing sessions in the form of network disruptions or user and researcher computer malfunctions. When such situations arose, the team prioritized the user’s comfort by reassuring the user and worked through solutions. This approach by the remote observers was essential to prevent users from being influenced by negative experiences that could bias their reactions toward the application.

At weekly meetings, the remote moderator and remote observers reported on user feedback to ML. Priority application refinements that would influence the design iteration before advancing to the next phase were identified for attention within 1 week.

### Data Management by the Remote Moderator and Silent Observers in the Broader Study

Once the testing session was done, the remote moderator immediately edited the Microsoft Teams transcripts and replaced the user’s name with a code number. Although the video quality for the recordings in Microsoft Teams was very good, the automatic transcription was challenging to work with in terms of occasionally getting wrong the order of who spoke, at times breaking up sentences in a way that was nonsensical, not always accurately capturing what the user was saying, and adding words not said or inserting odd words; for example, every time the user said “tag,” the transcription would show they said “take.” The remote moderator relied heavily on listening to the video-recorded session to decipher what the user was saying or referring to during the session. For example, if a user said, “I will click this button” in the transcript, the remote moderator was able to look back on the video and put an annotation in the transcript as to the exact button they clicked. The edited transcripts from the testing sessions were downloaded as a docx file and saved in the Microsoft Teams folder.

Some time was required by the remote moderator to become familiar with how to use the Qualtrics platform to capture project data. Creating the forms in Qualtrics was easy, as was editing them. However, linking separate forms proved to be challenging with the loss of consent forms and demographic data forms in phase 3. Both users in phase 3 had to complete the 2 forms again. The demographic data form and the usability tool were exported to respective csv files with the names and email addresses of the users removed. These csv files and consent forms (downloaded from Qualtrics) were uploaded for secure storage on ML’s system drive folder.

After each session, similar to the remote moderator, the remote observers relied on Microsoft Teams video recordings and transcripts to confirm their recorded values (eg, number of mouse clicks) on the performance metrics tool. The performance metrics tool was stored on Google Forms, and the videos were accessed by the remote observers from Microsoft Teams. Upon completion of the analysis, the performance metrics tool was uploaded and securely stored in ML’s system drive folder. Video recordings of the Microsoft Teams meetings were deleted after the transcripts had been edited, and the remote observers cross-checked their record keeping against the video-recorded sessions.

### Data Collection for This Case Study

ML circulated a written list of questions about the personal accounts and experiences of the lead remote moderator and 2 remote observers while executing their roles and responsibilities during the usability testing sessions (Multimedia Appendix 4 [6]). The list of questions was adapted from the questions by Wozney et al [6] used with moderators in their remotely moderated study. The remote moderator and remote observers submitted their written accounts to ML in response to each of the questions posed on the list.

## Results

### Overview

In response to the research question, the following is a narrative account of the respective role experiences of the remote moderator and the remote observers during the usability testing sessions. Their personal accounts are accompanied by reflections on their assigned tasks plus recommendations for the design of future usability testing sessions. ML collated and analyzed their written accounts. Content analysis was conducted, and the coded data that emerged were organized, synthesized, and interpreted as themes by ML [13]. ML’s analysis of the data was shared with the remote moderator and remote observers, who read the interpretation and contributed correct information or additional perspectives about their experiences.

The following are emerging themes from the analyses of the written accounts of the remote moderator and the remote observers: (1) dealing with personal stressors, (2) dealing with user anxiety, (3) maintaining social presence, (4) ethical response to the study protocol, and (5) communication during sessions.

### Themes

#### Dealing With Personal Stressors

The remote moderator experienced anxiety during the first few sessions that was greatly reduced in subsequent sessions. The initial anxiety was mainly due to having not yet established a comfortable flow of tasks; for example, it seemed that the initial users took too much time to read the training documents, which caused a sense of urgency in the remote moderator. However, the anxiety dissipated when the remote moderator grew to appreciate that, realistically, engaging with the educational training application and documents could take between 1 and 1.5 hours. She also found that working with Qualtrics was anxiety-provoking because of her limited experience and learning curve with the program. One mishap involved the loss of 2 signed consent forms because of the way the remote moderator set up the consent form in Qualtrics.

Cognitive overload for the remote moderator also occurred most often in the first few sessions. The remote moderator struggled to pay attention not only to what the users were doing but also to how to guide them when they became reliant on her for direction while also concentrating on their think-aloud responses. Future remote moderators should be well rested to better focus on conducting the interviews. It is also good to follow a script of user tasks with possible responses to anticipated user questions.

#### Dealing With User Anxiety

The remote moderator would start most sessions by chatting with and asking the user how their day was going, talking about a topic other than the testing session, or laughing at a shared joke. The occasional sidebar conversation occurred, which helped the users feel more comfortable during the testing session. During the course of the testing sessions, if the remote moderator detected that the user was feeling anxious, she would usually ask them about how they were feeling in that moment, and common user responses were “I am confused,” “I want to do good on this,” or “This is a lot of reading and I am a bit overwhelmed.” In addition to providing reassurance, the remote moderator made it clear that the users were not being tested but rather being asked to provide honest feedback about the application.

The early testing sessions involved users who would pause during the task without explanation. The remote moderator assumed that they were just resting and did not ask if they were confused. After the first 2 interviews, the remote moderator took a different, more validating approach that entailed instructing the user to explain what was on their mind or asking a specific question to clarify the user’s thoughts. The remote moderator would interject if the user was clearly not understanding how to complete a task (eg, when they tried to complete the tag multiple times, read the error message, and still did not understand how to tag instances in the timeline). Prompts by the remote moderator would be “What on this page do you want to click on?,” “Is there anything you are confused about on this page?,” or “What do you think you should do next?” Other times, it seemed that the user wanted direction from the remote moderator when they would say things such as “How do I do...” before engaging with the application on their own. In these situations, the remote moderator would try to remain impartial and respond with “What would you do if you were on your own right now and you had to figure that out?” Additional usual responses by the remote moderator were “What page on this website have you been to that might have that?” or “Maybe go read the documents again” instead of just directly telling them how to do it. However, if the user was struggling hard to figure something out, the moderator would tell them how to do it. This happened most frequently when users did not understand how to annotate (“tag”) the video when attempting to capture their inference of the dialogue partner’s thoughts or feelings.

#### Maintaining Social Presence

The remote moderator and remote observers found no ill effects in not being able to draw on in-person visual cues (eg, full body language) to provide expressive guidance to the user or for the remote moderator to gauge users’ responses to the application. Other visual cues were noticeable with screen sharing, especially the user’s facial reactions such as confusion with certain aspects of the application. Getting the user to think aloud was very helpful as they would narrate what action they were performing and the reasons behind it. The remote moderator consciously used her hands and facial expressions as visual cues for the user. By contrast, being in person could have made it easier for the remote moderator to explain where to locate something (eg, physically pointing and saying, “It is in the top left corner under the other button...no up a bit more”). Overall, there was no evidence of a negative impact of being physically distanced on the performance of the user.

The remote observers had limited social interaction with the users. However, toward the end of the testing sessions, the remote moderator would invite the remote observers to make some remarks to the user (eg, thank them for participating and share their appreciation for helpful feedback). This was important for rapport building, helped the user see how well they did during the session, and provided a last chance to offer added suggestions for application refinements to the remote observers. Otherwise, the remote moderator asked the remote observers to interject amid the testing session only if needed (eg, assistance with a task). A remote observer described that being remote allowed him to focus on record keeping without being distracted by the presence or actions of another person. This remote observer felt that, if user testing sessions were done in person, his own reactions or behaviors could potentially pose an undue influence on the user’s responses to interacting with the application.

#### Ethical Response to the Study Protocol

The remote moderator described how her virtual interaction with the users was likely different than it would have been if she had engaged in person with the users. The remote moderator felt obligated to work hard at making the user feel comfortable by consciously using verbal cues versus having a wide range of bodily cues to better express herself at her disposal. Users also required a lot of reassurance as they were conscious of their appearance and other visual cues (eg, facial expressions and body movement) while being video recorded. This might have made them more reticent to share their thoughts and feelings about the application. To foster user trust in the remote moderator and testing process, the remote moderator explained to the users before the scheduled session that 2 remote observers would be present who would have their cameras and microphones turned off while they took notes throughout the session. The remote observers would be invited to interact with the users before the end of the testing session.

#### Communication During Sessions

During the sessions, the remote moderator and remote observers used the private chat function in Microsoft Teams for impromptu requests such as requesting the remote moderator to ask the user to add questions or give feedback to one another without distracting the user. The remote observers would send the remote moderator a message if she forgot to do something, such as not starting the recording or forgetting to tell the user to do something. The chat function also helped the remote observers engage with the remote moderator in troubleshooting issues that arose during the testing sessions. At first, the remote moderator found the communication features in Microsoft Teams folders to be confusing. Sometimes, she would receive a notification from one of the remote observers and she would not know if it came from the chat box or a different communication channel in Microsoft Teams. Overall, the remote moderator and remote observers found that Microsoft Teams was a comprehensive platform with built-in features that fostered discrete and timely communication with each other during the testing sessions. After each testing session, Microsoft Teams made transcriptions readily accessible for the team to quickly analyze and then communicate in a timely manner to make joint decisions about priority application refinements.

Tangled conversations were common when the remote moderator and the user would speak over or interrupt each other. Other users would mumble or speak to themselves when using the application or reading the training documents. This caused issues for the remote moderator, particularly when she attempted to accurately discern their user experiences while editing the transcriptions. The users were simply asked to repeat themselves in live sessions whenever tangled or interrupted speech occurred. Overall, the remote moderator and remote observers felt that these issues were unique to remotely moderated testing sessions and required careful attention to promote clarity in user communication for quality audio capture and transcription.

## Discussion

### Principal Findings and Research Implications

Wozney et al [6] described that real-time usability testing involves human impact factors—often unpredictable—that can influence the quality of the testing session results. Extant literature provides only a rare glimpse into the influence of test environments on moderator and test user performances, which warrants further attention. Therefore, our aim was to contribute to the literature by providing further qualitative information for consideration in the design of future remote usability testing sessions. In this section, we discuss the main experiences encountered by the remote moderator and remote observers related to triangulating methods of data collection and using unfamiliar technology and software as well as managing personal stressors and user anxiety, maintaining social presence, and ensuring good lines of team communication during testing sessions. The recommendations identified in this section describe how the remote moderator and remote observers felt that emerging issues could be better addressed during testing sessions.

The usability methods used in this study were comparable with those used in related studies. In their recent scoping review, Maramba et al [23] found that 6 different usability methods were often used: quantitative methods using questionnaires and task completion and qualitative methods using think-aloud, interviews, focus groups, and heuristic methods. Our broader study saw that the remote moderator and remote observers used 4 of the 6 methods: a questionnaire, task completion, think-aloud, and interviews. The efforts of the remote moderator and remote observers corroborate those described by Maramba et al [23], where the use of the think-aloud protocol by developers led to further iterative application development (in comparison with the use of questionnaires, task completion, interviews, and focus groups). In total, 3 iterations of the application were informed by user feedback obtained in our pilot tests and from each phase of usability testing. In their review, Maramba et al [23] found that only 31.3% of the included papers reported having done at least one further iteration of the application. Although the think-aloud method might slow the process, it does not have a negative impact on the flow of user thoughts [22]. Tiriticco [24] described this method as simple, flexible, and affordable as well as not needing “special skills” or equipment. As in this and related studies, evidence suggests that the think-aloud process is an optimal method to garner user feedback for application refinement and ought to be the focus of training for remote moderators and observers.

When they described positive experiences, the remote moderator and remote observers said that they were primarily enabled by the functionality and features of the Microsoft Teams web conferencing technology and software with screen-sharing, video recording, and transcription features. Despite the initial lack of familiarity with the Microsoft Teams platform, the team quickly learned the technology because of their respective backgrounds in computer science and technology. Wozney et al [6] described that having to learn new web conferencing software can create a “cognitively demanding environment.” The team could have benefited from viewing demonstration videos or web-based tutorials about Microsoft Teams features (eg, chat and channel communication and the use of desktop and browser versions of the software) [7]. This would be in addition to running pilot sessions intended to clarify protocol steps and enable the coordination of team members in preparation for “real” testing sessions.

Technology also eased the cross-checking of the accuracy of record keeping as performed by the remote observers, who accessed the video-recorded sessions and transcriptions provided by Microsoft Teams. Furthermore, the use of Hotjar aided the remote observers in making more objective observations of how the user engaged with the application’s webpages (eg, user clicks, taps, scrolling behavior, and mouse movements) and identifying usability issues more easily. The triangulation of methods to capture user experiences (ie, video recordings, written transcriptions, and visualization of how the user maneuvered on the webpages) strengthened the reliability of the remote observers’ interpretation of user behavior [1]. This, in turn, led to their optimal decision-making regarding priority application refinements and rapid resolution of how to make application refinements before advancing to the next phase.

Regarding cognitive challenges, the remote moderator especially described her experience as stress-evoking. She needed to “take in” textual stimuli as well as auditory and visual stimuli while relying on her memory and interpretation skills as part of encouraging users to provide useful feedback for the remote observers and their application refinements [6]. Remote observers were less affected by cognitive overload and felt more efficient in their tasks. The lead remote moderator role was expected to not only learn the features and proficiently manage the functionality of Microsoft Teams in a short period (ie, during brief testing sessions) but also respond to anxious users’ questions, needs, and behaviors and to private chat room communication with remote observers during sessions. These expectations existed for the remote user who also needed to create a warm, inviting environment for users to provide honest responses to their experiences with the application. A recommendation to reduce the cognitive load for the lead moderator is to conduct pilot sessions with sufficient time for personnel to become familiar with testing demands and jointly strategize how to reduce anticipated cognitive challenges [22]. This would entail practice time to (1) learn advanced features in Microsoft Teams, such as the private chat function, to communicate with other team members; and (2) engage in rapport building with other team members (eg, silent observers) who will serve as a reassuring pair of added eyes and ears to address emerging issues during testing sessions. The other option is to hire experienced moderators and silent observers with advanced skills and knowledge of remote usability testing to efficiently deal with cognitive demands during sessions.

There were differences in opinion between the remote moderator and the remote observers regarding the benefits of in-person versus remote or virtual testing sessions in promoting social presence. Although the remote moderator had a larger role in interacting with the user, it is understandable that she felt constrained in not being able to provide more expressive instructions to the users—especially to users who were anxious when performing tasks. The remote observers did not feel hindered doing remote sessions. Rather, they felt that their use of screen sharing was sufficient for them to conduct silent observation and record keeping and not bias the user’s responses to the application. To overcome constrained communication with users, the remote moderator used the camera and audio features to maintain social presence (eg, with eye contact and body language) without feeling a need to talk more than necessary with the user. As described by Wozney et al [6], establishing and maintaining a social presence entails a combination of technical and interpersonal communication skills in the lead moderator. Practice exercises in virtual communication using technology and obtaining feedback from dialogue partners would be helpful in training research personnel in social presence.

Communication was an essential element that the team was keen to focus on to attain successful testing outcomes as fostered by technology. For instance, the private chat function in Microsoft Teams allowed for timely in-session communication and impromptu problem solving between the remote moderator and the 2 remote observers as required during the sessions. Being sensitive to the potential ill effects of physical distancing saw the team engage in careful efforts to create a friendly and accommodating environment for users [22]. For example, the remote observers were sensitive to their method of secretly communicating with the remote moderator during testing sessions and making themselves known to the user at the end of the session. The remote observers appreciated the opportunity to debrief with the user, express their gratitude, compliment the user for their efforts, and invite further user feedback. It is believed that these actions promoted trustworthiness in the testing experience and in the research personnel. Drawing on emerging evidence on establishing good web-based communication [22] would also be helpful in training remote moderators and silent observers.

### Limitations

A limitation related to “how” and “when” we captured the remote moderator’s and remote observers’ perceived experiences. First, the remote moderator’s and remote observers’ responses were based on their experiences with a small sample of nursing student participants from 1 setting, which poses a caveat to generalizing these findings to a wider student population. Second, their feedback was not solicited until after all usability testing sessions were completed and during a time when their student commitments were heavy. Furthermore, their narrative accounts may have been negatively affected by memory biases. A more reliable approach to capturing trustworthy accounts would have been to record a debriefing session individually with the remote moderator and remote observers immediately after each testing session. A focus group approach involving the remote moderator and remote observers could have also stimulated rich and more in-depth responses to their joint experiences in the testing sessions. The remote moderator and remote observers would need to feel comfortable enough with each other to honestly share how their collaborative efforts unfolded and were affected by each other’s behaviors.

This study’s main findings revealed a wealth of learning experiences by the remote moderator and remote observers in their respective roles when executing the usability testing protocol. Their use of web conferencing and survey technologies enabled adequate remote communication with users, good collaboration among research team members, the capture of user feedback, automatic transcription with immediate access for quick analysis, and easy remote administration of questionnaires. See Multimedia Appendix 5 for the remote moderator’s and remote observers’ recommendations relating to future usability testing sessions and a training protocol for research personnel.

### Conclusions

With the growing availability of web conferencing platforms, application developers are no longer restricted to in-person testing sessions. We anticipate a rising use of remote usability testing sessions as applications, services, technology, and software platforms continue to evolve and grow [23]. Usability testing is a relatively young science. This work contributes to the scarce literature on remote usability testing with web browser applications developed in academia, especially in health sciences [23]. “Best practice standards” are emerging where developers are required to publish evidence of user involvement and user satisfaction when seeking uptake of their application in the real world (eg, in clinical practice [25]).

Our results suggest that remotely moderated usability testing can serve as a valid substitute for traditional in-person usability testing. However, there remains a great need for more rigorous research to better comprehend the influence of remote moderator and silent remote observer characteristics, their previous experiences, and team collaboration on user responses. We also need more research that contributes evidence as a foundation for training remote moderators and remote observers in web-based interpersonal communication skills, the execution of usability testing protocols in virtual environments, team decision-making (eg, joint troubleshooting of emerging technical and user issues in “live” testing sessions), and technology and web conferencing technology skill proficiency. Finally, ongoing development and testing of reliable and valid methods to capture data remotely and record-keeping tools are warranted to ensure that rigorous studies are performed and outcomes are being captured to advance the science in usability testing. With such evidence in hand, videoconferencing software and technology developers can continue to create sound tools and methods for uptake by usability testing personnel.

## Appendix

Multimedia Appendix 1 Usability project setup.

Multimedia Appendix 2 Application development process.

Multimedia Appendix 3 Performance metrics tool.

Multimedia Appendix 4 Remote moderator and remote observer interview questions.

Multimedia Appendix 5 Recommendations for conducting future usability testing sessions and developing a training module.
