# Supplementary material for: Remote Moderator and Observer Experiences and Decision-making During Usability Testing of a Web-Based Empathy Training Portal: Content Analysis
Source: JMIR Form Res. 2022 Aug 3;6(8):e35319. doi: 10.2196/35319 (PMC9386579; doi:10.2196/35319)
Supplement: Multimedia Appendix 1 [file formative_v6i8e35319_app1.docx]

**Multimedia Appendix 1: Usability Project Set Up**

| Protocol Feature | What Phase? | 7Description |
| --- | --- | --- |
| Number of Phases (n = 3) |  | New groups of Years 2 to 4 undergraduate nursing student users for 3 phases |
| Number of Test Users (gender and year in nursing program) per Phase | Phase 1  Phase 2  Phase 3 | n = 3 (1 female, 4^th^ year; 1 male, 3^rd^ year; and, 1 female, 3^rd^ year)  n = 3 (1 female, 3^rd^ year; 1 male, 4^th^ year; and, 1 female 4^th^ year)  n = 2 (2 females, 4^th^ year) |
| Dates of Test Session | Phase 1 (3 user sessions)  Phase 2 (3 user sessions)  Phase 3 (2 user sessions) | July 15, July 20; and July 22  July 29; August 11; and, August 13  August 30 and August 31 |
| Number of remote moderators per Phase | Phases 1 to 3 | n = 1 |
| Number of silent observers per Phase | Phases 1 to 2  Phase 3 | n = 2  n = 0 |
| Access to intervention prior to remote usability session | Phases 1 to 3 | No |
| Software version | Phases 1 to 3 | MS Teams |
| Location of moderator per session | Phases 1 to 3 | Manitoba |
| Location of silent observers (x 2) per session | Phase 1  Phase 2 | India, Manitoba  India, Ontario |
| Tested training time for moderator and silent observers to set up sessions | Orientation to project including review of ethics documents, study protocol, study data collection tools, training in interviewing and moderating usability sessions; familiarization with the app and previous production (i.e., coding, scripting, and platforms); learning to work as a team during the pilot study including practice of protocol execution, joint review of user feedback followed by team decision-making with regard to priority app adjustments before real usability testing. | 50 hours |
| Location of test users | All 8 users | Manitoba |
| Moderator had prior experience moderating via web conferencing | Yes | No, not with MS Teams |
| Moderator had prior experience facilitating usability sessions | No |  |
| Moderator had prior experience facilitating research protocols | No |  |
| Silent observers had prior experience observing via web-conferencing | Yes | No, not with MS Teams |
| Silent observers had prior experience in record-keeping of user performance in sessions | Both | No |
| App adjusters had prior experience with development and/or fixing web-based applications | Both | Yes |
