# Supplementary material for: Remote Moderator and Observer Experiences and Decision-making During Usability Testing of a Web-Based Empathy Training Portal: Content Analysis
Source: JMIR Form Res. 2022 Aug 3;6(8):e35319. doi: 10.2196/35319 (PMC9386579; doi:10.2196/35319)
Supplement: Multimedia Appendix 2 [file formative_v6i8e35319_app2.docx]

**Multimedia Appendix 2: Development process here (programming language(s), IDEs and frameworks, code repositories and other tools.**

| **Git** | Version Control | General public license  Open source  Free  Off-the-shelf |
| --- | --- | --- |
| **Mongo DB Compass** | Database | BSD license  Proprietary  Free (12 months)  Off-the-shelf |
| **React** | Front-End | MIT License  Open source  Free  Off-the-shelf |
| **Node.js** | JS Runtime Environment | MIT License  Open source  Free  Off-the-shelf |
| **NPM** | Package Manager | Artistic license  Open source  Free  Off-the-shelf |
| **Python with Django** | Back-End | BSD license  Open source  Free  Off-the-shelf |
| **Amazon Web Services Simple Storage Service (Amazon S3)** | Video Storage | Server-side public license  Open source  Off-the-shelf |
| **Visual Studio Code** | IDE and Editor | MIT License  Open Source  Free |
| **Heroku** | Back-End Deployment |  |
| **Firebase** | Front-End Deployment |  |
