# Supplementary material for: Remote Moderator and Observer Experiences and Decision-making During Usability Testing of a Web-Based Empathy Training Portal: Content Analysis
Source: JMIR Form Res. 2022 Aug 3;6(8):e35319. doi: 10.2196/35319 (PMC9386579; doi:10.2196/35319)
Supplement: Multimedia Appendix 3 [file formative_v6i8e35319_app3.docx]

**Multimedia Appendix 3. Performance Metrics (n = 11 Tasks)**

| **Task** | **Indicators** |
| --- | --- |
| Create a New Account | Start Time  Number of Mouse Clicks to Finish Task  Completion Time  Successful Completion  Qualitative Comments  Error – Does not enter accepted email  Error – Fails to add comment to tagging  Qualitative comments |
| Log In | Start Time  Number of Mouse Clicks to Finish Task  Completion Time  Successful Completion  Qualitative Comments  Error – Does not enter accepted email  Error – Fails to Enter Correct Password  Qualitative Comments |
| Video Upload | Start Time  Number of Mouse Clicks to Finish Task  Completion Time  Successful Completion  Qualitative Comments  Error – Fails to Click on Correct Buttons or Links  Error – Fails to Upload the Video in the Required format  Error – Fails to Add Titles  Qualitative Comments |
| Creating a Tag | Start Time  Number of Mouse Clicks to Finish Task  Completion Time  Successful Completion  Qualitative Comments  Error - Fails to Click on Correct Buttons or Links  Error – Fails to Add Comment in Tagging  Qualitative Comments |
| Update an Existing Tag | Start time  Number of Mouse Clicks to Finish Task  Completion Time  Successful Completion  Qualitative Comments  Overall Comments/Remarks |
| Tagged Video Sharing | Start Time  Number of Mouse Clicks to Finish Task  Completion Time  Successful Completion  Qualitative Comments  Error – Fails to Enter Acceptable Email Address  Qualitative Comments |
| Exporting Tags in CSV format | Start Time  Number of Mouse Clicks to Finish  Completion Time  Successful Completion  Qualitative Comments  Error – Fails to Find the Correct Link  Qualitative Comments |
| Downloading Tagged Video | Start time  Number of Mouse Clicks to Finish  Completion Time  Successful Completion  Qualitative Comments  Error – Fails to Find the Correct Link  Qualitative Comments |
| Updating Information | Start Time  Number of Mouse Clicks to Finish  Completion Time  Successful Completion  Qualitative Comments  Error – Fails to Enter Required Information in Acceptable Format  Error – Fails to Enter Password  Qualitative Comments |
| Sign-out of logged account | Start Time  Number of Mouse Clicks to Finish  Completion Time  Successful Completion  Qualitative Comments |
| Forgot Password | Start Time  Number of Mouse Clicks to Finish  Completion Time  Successful Completion  Qualitative Comments  Error – Fails to Enter Acceptable Email Address  Error – Fails to Click on Correct Link or Button  Error – Is not able to enter the email address  Qualitative Comments  Overall Comments/Remarks |
