# Supplementary material for: Remote Moderator and Observer Experiences and Decision-making During Usability Testing of a Web-Based Empathy Training Portal: Content Analysis
Source: JMIR Form Res. 2022 Aug 3;6(8):e35319. doi: 10.2196/35319 (PMC9386579; doi:10.2196/35319)
Supplement: Multimedia Appendix 4 [file formative_v6i8e35319_app4.docx]

**Multimedia Appendix 4**

**Open-ended Questions for the Remote Moderator**

**Overarching Question: “What is the lead remote moderator’s experiences with the usability testing session protocols and tools employed?”**

**Instructions**: You can provide a bullet point list of steps you took in your role that I can write up. I realize your term is busy. If you could provide bullet point details such as any issues encountered and what you did to mitigate the issues. This type of detail would be helpful for future researchers and remote moderators.

**Here is a list of your assigned tasks you can write about.**

**1^st^ Invitations and Preparation**

- Describe what types of information were provided to student users in preparation for user testing sessions.
- Did you email them documentation in preparation for the session (e.g., user tasks, instructions on how to use MS Teams? What the app was about?, etc.)?
- Describe if you did a test run with the student users with MS Teams or anything else prior to the scheduled testing session? If not, do you recommend MS Teams for future researchers?

**2^nd^ Facilitating the Usability Testing Session**

- Describe whether you reminded the student user of the scheduled user testing session (e.g., the day before?). Any ‘no shows’ and attempts to get them to a scheduled session? Recommendations?
- Did you send a meeting invitation via MS Teams to the student user?
- Once the student user was logged in to the session, what did you do as the lead moderator? (e.g., address any issues). Describe anything of interest that would be helpful to future lead remote moderators)
- What was the computer technology skill level of the users? Did they use MS Teams before? What were the main issues and how did you help them? What were the most significant difficulties using MS teams by the student users (e.g., audio feedback, video, volume, clarity, software requirements, compatibility issues)?
- What did you use to guide the sessions (Phases 1 and 2) and Phase 3 (i.e., interview script, prompts, and tools)? Describe the tools you used as the lead moderator.
- Did you feel stressed during the sessions? Did you feel rushed to complete the usability protocol if more time was taken than anticipated? Was your own technical computer competence stressful (you needed more time to learn the ‘bells and whistles’ of using MS Teams or Qualtrics)?
- Did you experience cognitive load in terms of facilitating and observing the student users in testing sessions. This refers to dividing your attention between auditory, visual, and textual stimuli during the testing sessions creating “cognitive overload”. Did you experience this? What did you do (tips for future researchers)? Was this experienced more at Phase 1 but then it eased during subsequent Phases with increased familiarity with the protocol? Or did this happen with each user testing session? Paint a picture of your experience with detail and describe how you adapted to such a challenge.
- If you sensed that the user was stressed or anxious, what did you do? We must remember that student users had to deal with new technology and a novel application (cognitively demanding for them too!). Did you engage in laughing, light-hearted talk, or provide reassurances about technical prowess? In other words, how did you practice cognitive empathy or perspective-taking with the student users?

**3^rd^. Maintaining Social Presence during User Testing Sessions**

- As the lead observer what was your experience with the quantity and nature of social interactions or your ‘presence’? Did you have a desire to interject during the session or feel that you needed to wait for a prompt from the user to interact? Again, details are helpful for future researchers regarding any sense of negative outcomes because of physical disconnection.
- Did you have an ethical response toward the protocol that involved ‘silent’ remote observers? Did you tell the student users about them? What was their reaction to everyone observing them – especially toward the silent remote observers? Did you need to provide reassurances of their tasks and what information will be collected and how used?
- Describe if any and when direct communication occurred by you during the user testing sessions? What was the communication about and directed at whom? Was it good or negative for rapport-building? In other words, what was the outcome for you and the student user?
- Any technical challenges encountered such as tangled conversations (i.e., speaking over each other, non-intentional interrupting) or technical glitches?

**4th. Data Management**

- Describe your role and how it was done.
- You can provide details on how data was collected (e.g., video recordings were captured with MS Teams including closed captions that were automatically transcribed in MS Teams).
- Describe your experience using MS Teams to capture user feedback (e.g., needing to read and edit strange formatting and non-sensical words.
- What kind of clean-up was required (editing, reformatting)?
- Describe your experience using Qualtrics to capture data i.e., demographics and SUS.
- Describe data storage and accessibility to the usability session team and investigative team.

**5th. Other things to report about the videoconferencing environment and recommendations for future researchers?**

- You followed a script and a prompt list to get users to think aloud. Did you encounter any issues when doing that? If yes, what did you do to mitigate any issues encountered, for example, when the user did not think aloud, etc.).
- How did it feel for you to be remote from the user, the remote observers, and being mediated by the computer?
- Were you concerned about not having any in-person visual cues, such as body language to guide you? Do you think you behaved differently with the users when using the tool remotely than you would have in person?
- Do you think you captured different types of data using MS Teams via remote sessions than in-person? Describe in what way?
- Would you recommend using MS Teams to other researchers and remote moderators in conducting remote usability testing sessions? Why? Why not?

Adapted questions from Wozney et al. (2016).

**Open-ended Questions for the Remote Observers**

**Overarching Question: “What are the remote observer’s experiences with the usability testing session protocols and tools employed?”**

**Instructions**: I realize you term is busy. You can provide me with a bullet point list of steps you took in your roles doing ‘silent’ remote observation and app adjustments. Please provide me with details about steps taken, issues encountered, and what you did to mitigate the issues. This type of detail would be helpful for future researchers and remote observers and application adjusters.

**1^st^ Remote Quiet Observation and Tools employed**

- Describe what you did:
  - Did you need to be better prepared to help the lead moderator when issues arose during the sessions? Did you need to be better prepared or ‘at the ready’ to address anything (as described in Wozney et al., 2016) during the testing sessions?
  - How did you feel ‘ethically’ about being quiet remote observers? Did your feelings about this role influence your ability to observe and keep records? How did you manage those feelings if you had them about the silent observer role?
  - What environment did you work in as you remotely observed the testing sessions? Did this help you to control external stimuli that could distract your observation activities?
  - Seeing as we had 2 remote observers, did you use a private chat function with each other during the testing sessions for impromptu demands? Did you need to chat privately with the lead moderator during testing sessions or need to interrupt the session or did you wait until the session was done to give the lead moderator feedback? For example, did you need to help the lead moderator or the student users stay on task? In other words, what was the extent of your problem-solving required DURING user testing sessions.
- Describe the tools you created (e.g., the performance metrics tool with references)
- Describe other tools you employed (i.e., Hotjar)
- Describe your experiences using the respective tools
  - For example, with the Performance Metrics tool: Who used the tool, was the formatting good, the ease of use while observing, and any refinements required? Recommendations for future use?
  - I wonder if you encountered challenges with attempting to record user tasks in regard to communication issues that arose between the lead moderator and the student user (i.e., unclear information for you to record; dialogue jumping around and away from the task) and what did you do when that happened?
  - Hotjar heatmap – who used the tool, was it easy or challenging to use/interpret, how was it used as feedback for your refinements done to the app? Recommendations for future use?
  - Cognitive load (see Wozney et al., 2016, p. 6) in terms of managing the concurrent use of tools and observing student users in test sessions. I am referring to dividing your attention between auditory, visual, and textual stimuli during the testing sessions creating “cognitive overload”. Did you experience this? What did you do (tips for future researchers)? Was this experienced more at Phase 1 than in subsequent Phases with increasing familiarity in using the respective tools? Or did this happen with each user test session? Paint a picture of your experience with detail and describe how you adapted to such a challenge.

**2^nd^. Maintaining Social Presence during User Testing Sessions**

- As a ‘silent’ remote observer what was your experience with the quantity and nature of social interactions or your ‘presence’? Did you have a desire to interject during the session or felt you needed to wait for a prompt from the lead remote moderator? Did you have a protocol you followed to interact? Again, details are helpful for future researchers regarding any sense of negative outcomes because of physical disconnection.
- Describe (if any) direct communication that occurred by you with student users during the user testing sessions? What was the communication about and directed at whom? Was it good or negative for rapport-building? In other words, what was the outcome for you, the remote moderator, and the student user?
- Any technical challenges encountered (see Wozney et al., 2016, j. 7) such as tangled conversations (speaking over each other, non-intentional interrupting), or technical glitches (garbled audio or glitchy video-feeds).

**3^rd^ Data Management**

- Describe your role and how it was done.
- Did you need to clean up the data collected using performance metrics and Hotjar? What kinds of refinements were needed to the tool(s)?
- You can also provide details on how the data was and will be managed (stored) including who had access to it.
- Describe your experiences (good or negative) with data management, data sharing with other team members, and accessibility.

**4^th^. Other things to report about the videoconferencing environment and recommendations for future researchers?**

- Did you encounter any issues with task completion? If yes, what did you do to mitigate any issues encountered, for example, when the user did not think aloud, etc.).
- Details are good for future researchers of what you did, what worked, what didn’t, and what you did to overcome challenges (across all 3 Phases).

Adapted questions from Wozney et al. (2016).
