# Supplementary material for: Remote Moderator and Observer Experiences and Decision-making During Usability Testing of a Web-Based Empathy Training Portal: Content Analysis
Source: JMIR Form Res. 2022 Aug 3;6(8):e35319. doi: 10.2196/35319 (PMC9386579; doi:10.2196/35319)
Supplement: Multimedia Appendix 5 [file formative_v6i8e35319_app5.docx]

**Multimedia Appendix 5: Recommendations for conducting moderated role usability testing and training research personnel**

| **Activity** | **Tips** |
| --- | --- |
| **Conducting Usability Testing Session** | **Prior to the scheduled testing session**  **Research Personnel**   - Practice online communication techniques with a dialogue partner and seek feedback to improve technique - Coordinate tasks among the remote moderator and silent observers - Prepare recordkeeping tools and assign recordkeeping tasks - Select and practice with appropriate online collaborative document and behavior analytic tools (e.g., Google Documents, Hotjar) to capture user behavior - Select and practice with an appropriate communication tool (e.g., MS Teams private chat) for corresponding with research personnel during virtual sessions - Select and practice with an appropriate online survey platform (e.g., Qualtrics) to ensure proper linkage of study documents (e.g., consent form with online questionnaires) - Assign trained technical personnel to engage as both the silent remote observer and the app ‘fixer’ - Practice assigned tasks according to the testing protocol for team coordination - Be well rested before moderating and observing testing sessions   **User Participant**   - Send reminder emails 48 to 24 hours in advance of the session for the user to join the session and to download the web-conferencing application - Review materials, devices, environment, and technology needed (e.g., WiFi connection, audible mouse, quiet environment, web-conferencing application)   **Facilitating the session**   - Begin by thanking the user for participating and then review expectations for users in the session - Engage in friendly small talk before engaging in the recorded session - Remind the user to provide honest feedback to help with app fixes - Remind the user to open the same version (desktop or browser) of the web-conferencing application as the remote moderator - Explain how to share their screen before doing the tasks - Encourage the user to engage with the app as if they are alone - Remind users of the presence of silent remote observers who will have their cameras and mics turned off during the testing session - Continue to remind the user to speak out loud while they are doing tasks - Have a prompt sheet handy to use when encouraging users to do tasks on their own - Ask users to repeat themselves if they speak too softly or are unclear - Have a probe sheet handy in exit interviews when facilitating the think-aloud process with users - Provide reassurances to the user when technological glitches occur and that solutions will be found, jointly   **After the session**   - Allow silent observers to correspond with the user to express appreciation and seek further feedback if necessary - RM invites the user to provide any other feedback or concerns not already discussed - Thank the user participant - Check for the accuracy of transcriptions and recordkeeping against the video-recording. |
| **Training Research Personnel** | - Provide training in multiple methods for collecting user feedback and behaviors (questionnaires, task completion, think aloud process, and interviews or focus groups) - Hire experienced personnel in computer technology, web-conferencing software, and qualitative interviewing - Provide training in the use of web-conferencing and online survey software features and functionality (online tutorials, videos) - Provide a troubleshooting handbook of common usability testing issues with possible solutions - Do pilot tests to bolster familiarity with the testing protocol and coordinate tasks with other research personnel, and identify private communication techniques during sessions - Train in online communication and cultivation of social presence |
